# Loss of Diurnal Oscillatory Rhythms in Gut Microbiota Correlates with Changes in Circulating Metabolites in Type 2 Diabetic db/db Mice

**DOI:** 10.3390/nu11102310

**Published:** 2019-09-29

**Authors:** Eleni Beli, Samantha Prabakaran, Preethi Krishnan, Carmella Evans-Molina, Maria B. Grant

**Affiliations:** 1 Wellcome-Wolfson Institute for Experimental Medicine, Queens University Belfast, Belfast BT9 7BL, UK; 2 Department of Pediatrics, Indiana University School of Medicine, Indianapolis, IN 46202, USA; prekrish@iu.edu (P.K.); cevansmo@iu.edu (C.E.-M.); 3 College of Medicine, University of Central Florida, Orlando, FL 32827, USA; sprabakaran@Knights.ucf.edu; 4 Department of Ophthalmology, University of Alabama at Birmingham, Birmingham, AL 35233, USA

**Keywords:** circadian, type 2 diabetes, microbiota, metabolites, histidine, TMAO, methionine/cysteine, TCA cycle, urea cycle

## Abstract

Our hypothesis is that diabetes leads to loss of diurnal oscillatory rhythms in gut microbiota altering circulating metabolites. We performed an observational study where we compared diurnal changes of the gut microbiota with temporal changes of plasma metabolites. Metadata analysis from bacterial DNA from fecal pellets collected from 10-month old control (db/m) and type 2 diabetic (db/db) mice every 4 h for a 24-h period was used for prediction analysis. Blood plasma was collected at a day and night time points and was used for untargeted global metabolomic analysis. Feeding and activity behaviors were recorded. Our results show that while diabetic mice exhibited feeding and activity behavior similar to control mice, they exhibited a loss of diurnal oscillations in bacteria of the genus *Akkermansia*, *Bifidobacterium*, *Allobaculum*, *Oscillospira* and a phase shift in the oscillations of *g.Prevotella*, proteobacteria, and actinobacteria. Analysis of the circulating metabolites showed alterations in the diurnal pattern of metabolic pathways where bacteria have been implicated, such as the histidine, betaine, and methionine/cysteine pathway, mitochondrial function and the urea cycle. Functional analysis of the differential microbes revealed that during the day, when mice are asleep, the microbes of diabetic mice were enriched in processing carbon and pyruvate metabolic pathways instead of xenobiotic degradation as was observed for control mice. Altogether, our study suggests that diabetes led to loss of rhythmic oscillations of many gut microbiota with possible implications for temporal regulation of host metabolic pathways.

## 1. Introduction

The number of adults with type 2 diabetes has quadrupled, in part, because of the rise in obesity and an increase in the aging population [1]. Diabetes and metabolic dysfunction adversely impacts the diurnal rhythms of rest and activity, body temperature, hormone secretion, and gene expression [2,3]. The molecular machinery of circadian rhythms consists of a tight loop of expression and degradation of transcription factors [4] regulated by signals generated in the suprachiasmatic nucleus (SCN), such as hormones and neuronal activity [2,5,6], and also by signals generated in the periphery, from feeding and activity behavior [7]. We and others have observed that diabetic individuals and diabetic rodents exhibit altered circadian rhythms [3,8,9,10]. Indeed recently, insulin was identified as a direct driver of the expression of the circadian gene, Per2, and mis-timed insulin signaling disrupted the circadian regulation and clock gene expression [11]. Altered circadian rhythms in diabetes facilitate the progression of vascular complications. We have shown that deletion of circadian genes, by mutation of Per2 in mice [12] or by deletion of Bmal-1 in endothelial cells [13] results in a vascular phenotype similar to diabetic retinopathy (DR) [12].

Apart from the role of altered gut microbiota composition in diabetes [14,15], a growing body of evidence supports that gut bacteria have their own circadian rhythms [16,17,18] affecting the host metabolism [19,20,21]. Bacteria rhythmically adhere to the colon exhibiting oscillatory metabolic activity and provide intermediate metabolic byproducts that regulate diurnal functions of colonic cells [21]. Bacterial pathways that exhibit diurnal regulation include nucleotide metabolism, amino acid metabolism, and mucus degradation [17]. During the night when mice are active, pathways involved in bacterial energy metabolism, DNA repair, and cell growth are found to be increased. During the day, when mice are asleep, pathways involved in detoxification, motility, and environmental sensing (flagellar assembly, bacterial chemotaxis, and type III secretion) are enriched [17]. When the host is actively feeding, as in the night for mice, gut bacteria utilize nutrients for cell growth, but when the host is asleep, as in the day for mice, digested food is absent and bacteria mobilize to penetrate the mucus layer and utilize mucins or host metabolites as nutrients [22]. Whether type 2 diabetes affects the circadian regulation of the gut microbiota is not known. Furthermore, the implications of altered gut microbiota oscillations for the host metabolism are incompletely understood.

In the current study, we put forth the hypothesis that type 2 diabetes would alter the rhythmic oscillations of the gut microbiota impacting the host metabolism. We performed an observational study to compare the diurnal changes of the gut microbiota with temporal changes of the plasma metabolites. The study was designed as a metadata analysis of the microbiota data from a previous study of 10-month ad-lib db/db and db/m mice [23] with the incorporation of global metabolomics of peripheral blood. Our study identified in the diabetic mice a loss of diurnal oscillations in many genera of the gut microbiota. Analysis of the circulating metabolites showed alterations in the diurnal pattern of metabolites in the histidine pathway, loss of the diurnal pattern in metabolites involved in betaine and methionine/cysteine pathway with implications for increased TMAO production. Moreover, diabetic mice exhibited a gain in the diurnal pattern of the TCA and urea cycle. Gut microbiota are implicated in these metabolic pathways. Moreover, our functional analysis of the differential microbes revealed that during the day, when mice are asleep, the microbes of diabetic mice were enriched in pathways involved in carbon and pyruvate metabolism instead of xenobiotic degradation as in controls, although diabetic mice did not have different feeding or activity behavior. Altogether, our studies suggest that tyep2 diabetes led to a loss of rhythmic oscillations of many gut microbiota with possible implications in the temporal regulation of metabolic pathways.

## 2. Materials and Methods

### 2.1. Experimental Design

Male B6.BKS(D)-Lepr^db^/J (stock number:000697) homozygous db/db mice and heterozygotes (db/m) were used as the diabetic and control mice. All mice were obtained from Jackson Laboratory (Bar Harbor, ME) and housed in the institutional animal care facility at the Indiana University (IACUC #10604 and #11167). Animals were fed ad libitum for 10 months with chow diet (2018SX, Harlan, 18% protein, 5% fat, 5% fiber). We obtained fecal samples every 4 h within a 24-h period and plasma samples at ZT5 and ZT17. Zeitgeber time (ZT) indicates the time when the lights are on. Mice, as nocturnal animal, have a “rest phase” when the lights are on, from ZT0 to ZT12 and an “active phase” when the lights are off from ZT12 to ZT0.

### 2.2. Microbiota Analysis 

The data used for the microbiota analysis were extracted from the dataset first published in *Diabetes* [23]. The microbiota data are uploaded in ENA under the project name “fecal db/db Intermittent Fasting.” The data used in this study are only from the ad-libitum groups.

### 2.3. Statistical Analysis

The data were plotted as mean ± SEM. The diurnal data represents time series data and cosine analysis was employed to fit the data into a smooth curve. The data was considered as diurnal oscillation by the zero-amplitude test with a *p*-value of less than 0.05. The expression level between different age groups was compared using two-way ANOVA with Tukey post-hoc analyses (GraphPad Prism 8, San Diego, CA, USA).

### 2.4. Prediction Analysis

We used Piphillin, a software that predicts metagenomes using direct nearest- neighbor-matching between 16S rRNA amplicons and genomes to predict the represented genomes [24]. List of the deferential expressed bacterial KO pathways identified using the DESeq2 package [25] were used with MicrobiomeAnalyst for pathways analysis [26].

### 2.5. Global Metabolomic Analysis

The samples used for this manuscript were extracted from the same animals used for the microbiota analysis [23]. Whole blood was collected at the termination of the experiment by cardiac puncture at 10 months of age at two time points: one during the day (ZT5) and one during the night (ZT17). Blood was collected in EDTA tubes and plasma separated and frozen at −80 °C. Samples were sent to Metabolon Inc (Morrisville, NC, USA) and global metabolic analysis was performed with their Metabolon Platform using a Waters ACQUITY ultra-performance liquid chromatography (UPLC) and a Thermo Scientific Q-Exactive high resolution/accurate mass spectrometer interfaced with a heated electrospray ionization (HESI-II) source and Orbitrap mass analyzer operated at 35,000 mass resolution. Samples were prepared using the automated MicroLab STAR^®^ system from Hamilton Company. Several recovery standards were added prior to the first step in the extraction process for QC purposes. Proteins were precipitated with methanol under vigorous shaking for 2 min (Glen Mills GenoGrinder 2000) followed by centrifugation. The resulting extract was divided into five fractions: two for analysis by two separate reverse phase (RP)/UPLC-MS/MS methods with positive ion mode electrospray ionization (ESI), one for analysis by RP/UPLC-MS/MS with negative ion mode ESI, one for analysis by HILIC/UPLC-MS/MS with negative ion mode ESI, and one sample was reserved for backup. Samples were placed briefly on a TurboVap^®^ (Zymark) to remove the organic solvent. Several controls were analyzed in concert with the experimental samples: a pooled matrix sample generated by taking a small volume of each experimental sample served as a technical replicate throughout the data set; extracted water samples served as process blanks; and a cocktail of QC standards that were carefully chosen not to interfere with the measurement of endogenous compounds were spiked into every analyzed sample, allowed instrument performance monitoring and aided chromatographic alignment. The description of laboratory information management system (LIMS), the data extraction and peak-identification software, data processing tools for QC and compound identification, and the procedures of collection of information interpretation and visualization tools are described in detail in the Supplemental Material. Two-way ANOVA was used to identify statistically significant differences between the control and diabetic and between day and night. Lists of statistically significant differential metabolites were used with the MetaboloAnalyst 4.0 [27] for pathway, enrichment analysis, principle component analysis (PCA) and generation of heatmaps. The metabolomics data can be found at ftp://www.metabolomicsworkbench.org under the project “db/db plasma Day vs. Night.”

### 2.6. Activity and Food Consumption

Measurements were performed using a TSE systems LabMaster Metabolism Research Platform (Chesterfield, MO) equipped with calorimeter, feeding, and activity system. All measurements were performed after a 48-h acclimation period followed by 48-h of data collected every 10 min and results are displayed in terms of mean measurements over a 24-h period as well as during light (0700–1900) and dark cycles (1900–0700).

### 2.7. Gene Expression Analysis

Colon tissue was extracted at the termination of the experiment and preserved in RNAlater solution (Invitrogen Inc, Waltham, MA, USA) overnight at 4 °C and then at −80 °C until processing. RNA extraction was performed with homogenization and TRIzol reagent (Invitrogen Inc,). A total of 1000 ng RNA was used for reverse transcription using the Vilo cDNA Synthesis Kit (Invitrogen). Primers for *Arnl-1* and *Per-2*, were purchased from Thermo Scientific/Applied Biosysems (Carlsbad, CA, USA) and qRT-PCR was carried out using TaqMan master mix (Applied Biosystems) on a ViiA7 RT-PCR system with a 384 well block.

## 3. Results

### 3.1. Diurnal Rhythmic Oscillations of the Gut Microbiota in Type 2 Diabetic Mice

**Figure 1:**
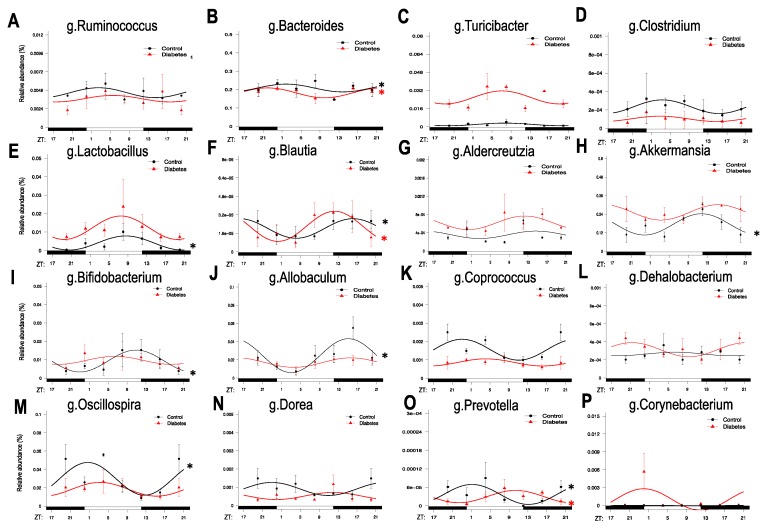
Diurnal oscillatory rhythms of the gut microbiota at the genus level in type 2 diabetes. Bacterial 16SrRNA sequence analysis was performed in fecal samples from 10 month old db/db (red) and aged-matched controls, db/m (black) collected every 4 h for a 24-h period at ZT0, ZT4, ZT8, ZT12, ZT16, ZT20. ZT indicates zeitgeber time, i.e., hours after the lights are on. (**A**–**P**) Identified OTUs belonging to each genus were summed up and cosinor analysis was performed for each genus. *n* = 3 per time point per group. Asterisks indicate the genera that exhibit significant oscillatory rhythmicity using the zero -amplitude test with a *p*-value of less than 0.05.

We used the sequences identified from the microbiota study published by Beli et al. 2018 for the group of ad-libitum fed mice [23]. In that study, we identified 109 different species of bacteria in control and diabetes on ad-libitum feeding with increased *Corynobacterium*, *Allobaculum*, *Aerococcus*, *Turibacter*, *Lactobacillus*, *Coprobacillus* and a reduction in *Ruminococcus*, *Oscillospira*, *Coprococcus*, and *Roseburia* in diabetic mice [23]. In the current study, we reanalyzed the OTUs using the cosine function at the genus (Figure 1) and family level (Supplemental Figure S1) rather than the OTU level. We focused on the genera identified to be more abundant and different between the control and diabetic mice [23].

A general observation is that different genera peaked at different times during the 24-h period, with some genera—at least in the control mice—peaking at the switch from night to day, others during the day, when mice are asleep and no longer feeding, others at the switch from day to night in anticipation of feeding, and others during the late night, when mice were actively eating. In the control mice, bacteria that were increased during the end of the night and peaked at the beginning of the day period belonged to the genera of *Ruminococcus* (Figure 1A) and *Bacteroides* (Figure 1B). Bacteria that peaked later during the day, when mice were asleep and inactive, belonged to the genera of *Turicibacter* (Figure 1C), *Clostridium* (Figure 1D), and *Lactobacillus* (Figure 1E). Bacteria that peaked at the switch from day to night, belonged to the genera of *Blautia* (Figure 1F), *Adlercreutzia* (Figure 1G), *Akkermansia* (Figure 1H), *Bifidobacterium* (Figure 1I), and *Allobaculum* (Figure 1J). Finally, bacteria that peaked later toward the end of night, when feeding was completed, belonged to the genera of *Coprococcus* (Figure 1K), *Dehalobacterium* (Figure 1L), *Oscillospira* (Figure 1M), *Dorea* (Figure 1N), *Prevotella* (Figure 1O), and *Corynobacterium* (Figure 1P).

Bacteria of the genus *Bacteroides* (Figure 1B), *Lactobacillus* (Figure 1E), *Blautia* (Figure 1F), *Akkermansia* (Figure 1H), *Bifidobacterium* (Figure 1I), *Allobaculum* (Figure 1J), *Oscillospira* (Figure 1M), and *Prevotella* (Figure 1O) showed strong diurnal oscillations in control mice, but in diabetic mice only the genus *Bacteroides, Blautia*, and *Prevotella* preserved their diurnal rhythmicity, while the rest of the bacteria had reduced amplitudes or lost circadian regulation entirely. Interestingly, in diabetes, some bacteria exhibited a phase shift in their peak levels compared to controls. For example, a phase advancement for *Blautia* (Figure 1G) and a phase delay for *Prevotella* (Figure 1O) was observed.

At the family level, the gut microbiota of diabetic mice (Supplemental Figure S1A) showed a total loss of oscillatory rhythmicity in the families of Lachnospiraceae, Erysipelotrichaceae and Bifidobacterium, blunted oscillations for the families of Ruminococcaceae, Lactobacillaceae and Verrucomicrobiaceae, and a phase shift in the family of Prevotellaceae. Further effects on gut oscillations were also observed at the class level (Supplemental Figure S1B). Proteobacteria in general exhibit very weak rhythmic oscillations, but in diabetic mice they peaked during the night while in controls they peaked during the day. While Clostridia and Coriobacteria maintained their rhythmic oscillations, Actinobacteria and Erysiplotrichi lost their rhythmic oscillations. Bacilli and Verrucomicrobia (mostly *Akkermansia municiphila*) are enriched with diabetic mice but lost their rhythmic oscillations. At the phylum level (Supplemental Figure S1C), Firmicutes maintained oscillations in diabetic mice, but Actinobacteria and Proteobacteria exhibited shifts in the time of their peak expression. Remarkably, Bacteroidia did not exhibit any diurnal rhythmicity.

### 3.2. Diurnal Patterns of Blood Metabolome in Type 2 Diabetic Mice

To correlate the loss of circadian rhythmicity in the microbiota of diabetic mice with possible metabolic pathways, we performed a global metabolomics analysis of blood samples from our cohorts collected at different times of the day. The goal was to identify metabolites and their associated pathways that were altered in type 2 diabetes and to examine if there was a possible role of the microbiota in the identified metabolic pathways based on known and predicted microbiota functions. Since the microbiota oscillations did not have a single peak or trough time, but rather their peaks occurred throughout the 24-h cycle, we chose two representative time points for the metabolomics analysis: one during the night, ZT17, when the mice were active and feeding, and one during the day, ZT5, when mice were sleeping. Table 1 summarizes the statistical analysis (one-way ANOVA) of the identified metabolites in the plasma. Overall, 747 biochemicals were detected and 294 were changed because of diabetes, while 178 were changed because of a diurnal effect (day vs. night).

**Figure 2:**
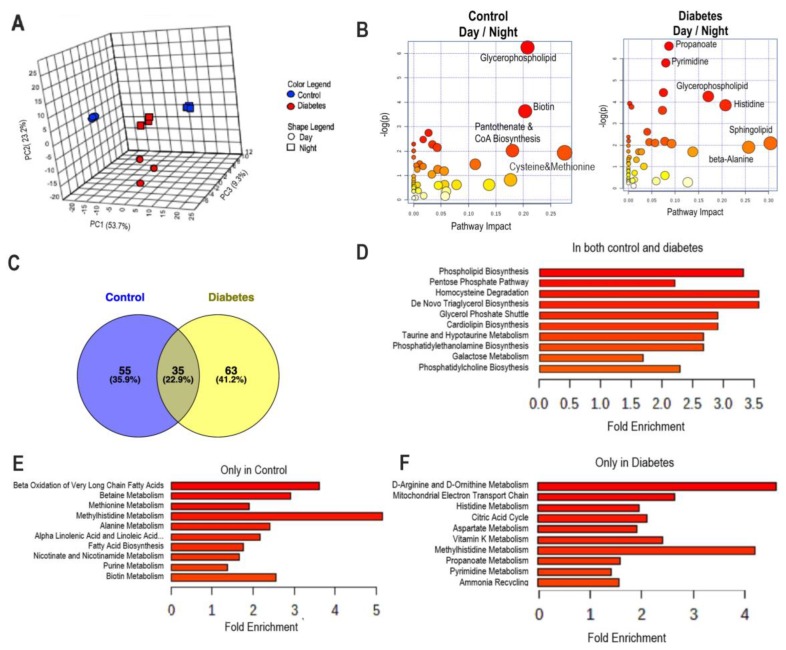
Global metabolomic analysis of plasma metabolites. Blood samples were collected at ZT5 (day) and at ZT17 (night) from 10-month-old db/db (diabetic) and aged-matched db/m (control) mice. Untargeted global metabolomics analysis was performed with the Metabolon Platform (Metabolon Inc). Statistically significant different metabolites were used for the analysis with MetaboAnalyst. (**A**) PCA analysis. (**B**) Metabolic pathway analysis of metabolites that were different in the transition from day to night in control and diabetic groups. (**C**) Venn graphs of metabolites that were different in the transition from day to night in control and diabetic groups. (**D**) Enrichment analysis of metabolites that similarly changed in the transition from day to night in control and diabetic groups. (**E**) Enrichment analysis of metabolites that changed in the transition from day to night only in control. (**F**) Enrichment analysis of metabolites that changed in the transition from day to night only in diabetes. *n* = 3 per time point per group.

While there was an equal number of metabolites that changed in day vs. night in both cohorts (control and diabetes; 113 vs. 130), PCA analysis revealed that the metabolites found in the day were clearly separated from those found in the night in both the controls and the diabetic mice (Figure 2A). In controls, differential metabolites (day vs. night) were separated based on PC1 (53.7%). In diabetic mice, differential metabolites were separated based on the PC2 (23.3%) and not on PC1, indicating different components were responsible for this variability during the transition from day to night in diabetes vs. controls (Figure 2A). Then, we performed pathway analysis on the metabolites in both cohorts focusing on differential metabolites in day vs. night (Figure 2B). Plasma from control mice collected during the day when compared to night was enriched in metabolites belonging to pathways of glycerophospholipid, biotin, pathothenate/CoA biosynthesis, and cysteine/methionine metabolism pathways. Plasma from diabetic mice collected during the day when compared to night had metabolites from the glycerophospholipid pathway, and in addition from the pathways of propanoate, pyrimidine, histidine, sphingolipid, and beta alanine metabolism.

Venn diagrams were used to identify the unique and common metabolites that were different at the transition from day to night in control and diabetic mice (Figure 2C). While we found that there were shared metabolites in both cohorts (Figure 2C,D), the majority of metabolites that changed during the transition from day to night were unique to each cohort. Enrichment analysis revealed that metabolites that changed from day to night in the control were associated with beta oxidation of the very long-chain fatty acids, betaine, and methionine metabolism (Figure 2E), whereas in diabetic mice the metabolites were associated with D-amino acid metabolism, mitochondrial function, energy production, and histidine metabolism (Figure 2F).

**Figure 3:**
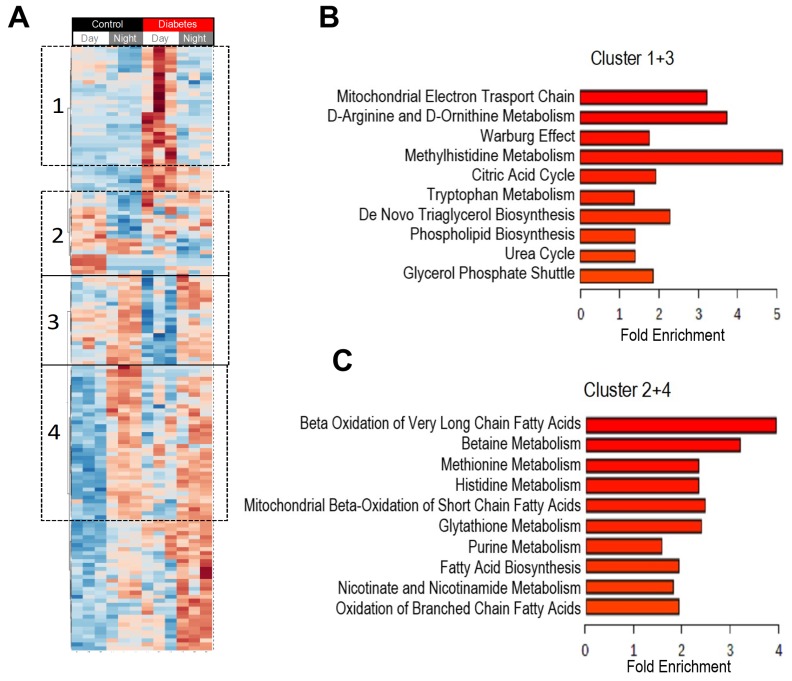
Heatmap of plasma metabolites that changed during the transition from day to night. Blood samples were collected at day time (ZT5) and at night time (ZT17) from 10-month-old db/db (diabetic) and aged-matched db/m (control) mice. Untargeted global metabolomics analysis was performed with the Metabolon Platform (Metabolon Inc). MetaboAnalyst was used for the analysis. (**A**) Heatmap of statistically significant metabolites. We identified clusters that gained diurnal rhythmic patterns (increased (Cluster 1) or decreased (Cluster 3) in diabetes in the day but did not change in controls and clusters that lost diurnal rhythmic patterns in diabetes (increased (Cluster 2) or decreased (Cluster 4) in control in the day but did not change in diabetes. (**B**) Enrichment analysis of metabolites that gained rhythmic patterns in diabetes (Cluster 1 and 3). (**C**) Enrichment analysis of metabolites that lost rhythmic patterns in diabetes (Cluster 2 and 4). *n* = 3 per time point per group.

To identify if there were specific patterns in how the metabolites changed from day to night in control and diabetic mice, we generated a heatmap of plasma metabolites that were significantly different from day to night and between control and diabetes (Figure 3A). The heatmap revealed clusters of metabolites that exhibited a diurnal pattern only in diabetes (Cluster 1 and 3) and not in controls, and clusters of metabolites with a clear diurnal pattern in controls but not in diabetes (Cluster 2 and 4). Enrichment analysis of the metabolites identified in clusters that “gained” a diurnal pattern in diabetic mice (Clusters 1 and 3) indicated enrichment into pathways of the mitochondrial electron transport, D-amino acids, glycolysis, methylhistidine metabolism, TCA cycle, and urea cycle among others (Figure 3B). Metabolites identified in the clusters that lost a diurnal pattern in diabetic mice (Clusters 2 and 4) belonged with pathways of beta oxidation of very long-chain fatty acids, betaine, methionine, histidine metabolism, and beta oxidation of short-chain fatty acids (Figure 3C).

### 3.3. Histidine, Betaine, and Cysteine/Methione Pathway in Type 2 Diabetes

**Figure 4:**
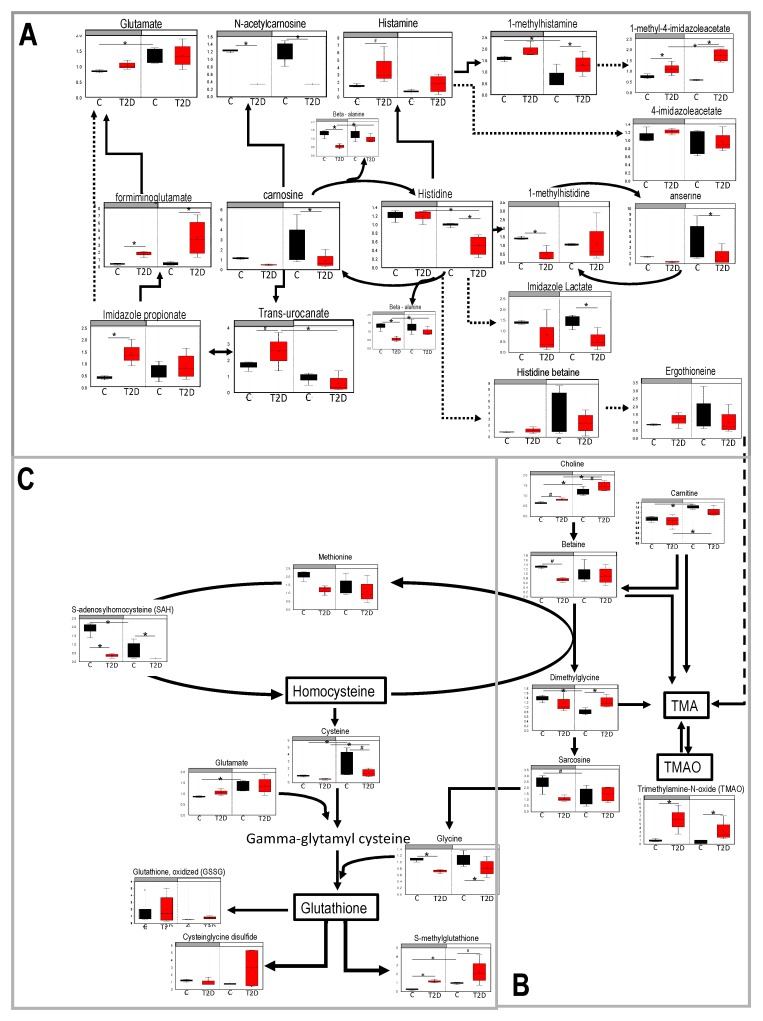
Pathways of metabolites that showed up in the cluster that lost diurnal patterns in type 2 diabetes (T2D). Blood samples were collected at day time (ZT5) and at night time (ZT17) from 10-month-old db/db (diabetes, red) and aged-matched db/m (control, black) mice. Untargeted global metabolomics analysis was performed with the Metabolon Platform (Metabolon Inc). (**A**) Histidine metabolism pathway. (**B**) Betaine metabolism pathway. (**C**) Methionine/cysteine pathway. *n* = 3 per time point per group. Two-way ANOVA. (*) indicates statistical difference *p* < 0.05, and (#) *p* < 0.01 in post-hoc comparisons. y-axis shows the volume and median = 1 normalized values of area under the curve values form each metabolite. Bars above each chart (grey vs. white) indicate night vs. day.

From the above analysis, we observed that diabetic mice exhibited a loss of diurnal rhythmicity in pathways involved in the antioxidant cysteine/methionine, betaine metabolism, and beta oxidation of very long and short-chain fatty acids, while there was a gain of rhythmicity in pathways involved in mitochondrial function and the urea cycle. Interestingly, metabolites that belonged to the histidine pathway were among those who both gained and lost rhythmicity in diabetes. We interrogated the above pathways to determine the specific microbial metabolites generated. Figure 4A shows the identified metabolites involved in the histidine pathway and its interception with the betaine (Figure 4B) and cysteine/methionine pathway (Figure 4C).

The histidine metabolic pathway was altered in diabetes as several acetylated and methylated histidine derivatives were different between the control and diabetic groups (formiminoglutamate, imidazole propionate, urocanate, carnosine, *N*-acetylcarnosine, histamine, methylhistamine, 1-methyl-4 imidazoleaceetate, beta alanine, histidine, and imidazole lactate) (Figure 4A). Some metabolites gained a diurnal pattern in diabetes (i.e., they were different from day to night in diabetic but not in control mice), such as (urocanate, 1-methyl-4 imidazoleaceetate, beta alanine, histidine) and other metabolites lost a diurnal pattern (1-methylhistamine, glutamate). Interestingly, imidazole propionate, a microbial metabolite that has been linked to insulin resistance [28] was increased during the night in diabetic mice instead of following the trend as in controls which was toward an increase during the day. Formiminoglutamate, which is produced from imidazole propionate was also significantly elevated in diabetes. Histidine showed a significant reduction from day to night and histamine and its derivatives demonstrated an increase in the diabetic mice. Ergothioneine, another microbial byproduct that is also involved in the production of trimethylamine (TMA) and trimethylamine *N*-oxide (TMAO) (Figure 4B) was detected, but no differences were observed. Finally, beta alanine, was significantly reduced in diabetes. Reduced beta alanine leads to reduced carnosine and anserine, two dipeptides that can act as antioxidants [29,30,31].

The betaine metabolic pathway (Figure 4B) also appeared to be altered in diabetes. Choline and carnitine preserved their diurnal change but in the night there was more choline and less betaine in diabetes (Figure 4C). Betaine and dimethylglycine lead to synthesis of TMA and TMAO, which are of particular interest because they are associated with cardiovascular diseases and require intestinal bacteria for their synthesis [32]. Plasma of diabetic mice showed elevated the TMAO levels in both day and night (Figure 4B) implicating that the gut microbiome composition may be responsible for this change. Moreover, sarcosine (Figure 4B) which is degraded to glycine and affects glutathione synthesis lost its diurnal rhythm.

The methionine/cysteine metabolic pathway (Figure 4C) was identified in the cluster of metabolites that lost diurnal change in diabetic mice. While S-adenosylhomocysteine (SAH) exhibited a clear loss of diurnal change in diabetes, methionine showed only a trend toward loss. Cysteine showed a smaller degree of change from day to night in diabetic mice. Finally, we observed that oxidized glutathione, cysteine-glutathione disulfide, and S-methylglutathione were higher (or tended towards higher) in diabetic mice, consistent with the observation that diabetes is associated with elevated oxidative stress.

### 3.4. Glucolysis, TCA, and Urea Cycle in Type 2 Diabetes

**Figure 5:**
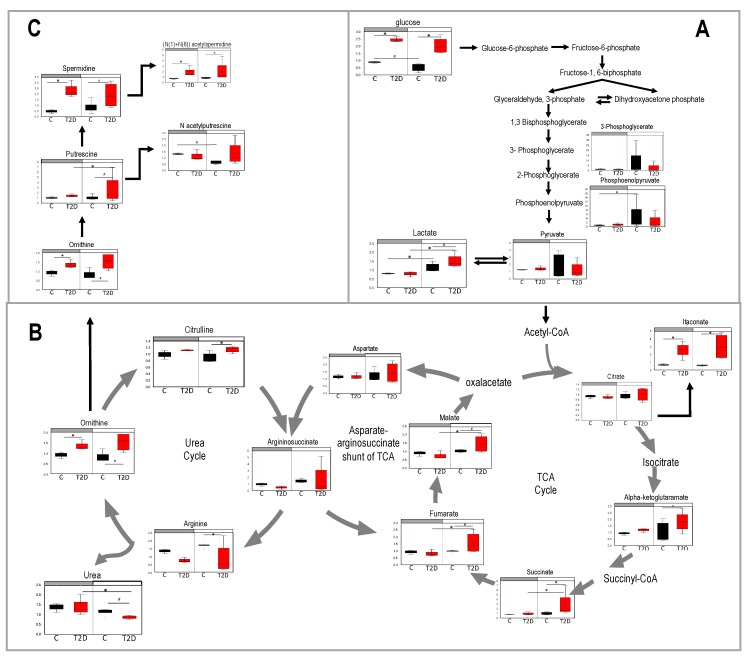
Pathways of metabolites that showed up in the cluster that lost diurnal patterns in type 2 diabetes (T2D). Blood samples were collected at day time (ZT5) and at night time (ZT17) from 10-month-old db/db (red) and aged-matched db/m (black) mice. Untargeted global metabolomics analysis was performed with the Metabolon Platform (Metabolon Inc). (**A**) Glycolysis, (**B**) TCA cycle and urea cycle, and (**C**) polyamine metabolism. *n* = 3 per time point per group. Two-way ANOVA. (*) indicate statistical difference *p* < 0.05, and (#) *p* < 0.01 in post-hoc comparisons. y-axis shows the volume and median = 1 normalized values of area under the curve values form each metabolite. Bars above each chart (grey vs. white) indicate night vs. day.

The heatmap in Figure 3B revealed that metabolites that gained a diurnal pattern in diabetic mice belonged to pathways involved in the Warburg effect, mitochondrial electron transport, TCA, and urea cycle. Metabolites involved in glycolysis and the Warburg effect are shown in Figure 5A.

Glucose was elevated in both day and night in diabetic mice and lactate exhibited a robust diurnal pattern with a significant increase in diabetes. The TCA cycle and its interception with the urea cycle is depicted in Figure 5B. Metabolites of the TCA cycle that gained a diurnal pattern were succinate, fumarate, and malate. Succinate and fumarate are also part of the mitochondrial electron transport pathway. These metabolites were significantly elevated during the day in diabetes when mice were asleep. Itaconate and alpha-ketoglutaramate were also significantly elevated in diabetic mice but they did not exhibit a diurnal pattern.

The urea cycle was also among the metabolites that gained a diurnal pattern in diabetes. Urea showed a significant depletion during the day in diabetic mice. Yet, arginine, ornithine, and citrulline were elevated in diabetic mice, especially during the day. Finally, we examined the polyamine metabolism pathway (Figure 5C) as Thaiss et al. [21] had identified this pathway as a target of the oscillatory gut microbiota. Again, we saw elevated levels of polyamines in diabetic mice and putrescine gained a diurnal pattern.

### 3.5. Prediction Analysis of Durnal Patterns in Bacterial Functions

Although we identified a loss of bacterial oscillations and many metabolites that either gained or lost a diurnal pattern in the blood, our studies do not establish that the changes in the blood are caused by the loss of circadian regulation in the microbiota. To define a possible relationship between the microbiota and the metabolites in the blood, we performed a prediction analysis of pathways identified from 16SrRNA in our cohorts (Table 2). This analysis provides predicted metabolic functions for the microbiota. We only used two-time points, ZT5 and ZT17 in order to match the microbiota time points with the time points of the plasma samples. During the day, when mice are asleep and are not feeding, the bacterial-predicted functions in control mice included zeatin biosynthesis and xenobiotic degradation. In contrast, in diabetes, the pathways the bacterial-predicted functions were carbon and pyruvate metabolism consistent with higher energy production when mice should have been asleep. These data suggest that type 2 diabetes not only results in loss of diurnal oscillations in the microbiota taxa but also in the functional output of the microbiota. Yet, this analysis still does not establish that the changes observed in the blood are due to the temporal changes in the microbiome, it suggests that the diabetic microbiota are actively metabolizing and growing during the day, when mice are asleep and not fed, and that they can possibly affect the host metabolism.

### 3.6. Circadian Rhythms in Type 2 Diabetic Mice

**Figure 6:**
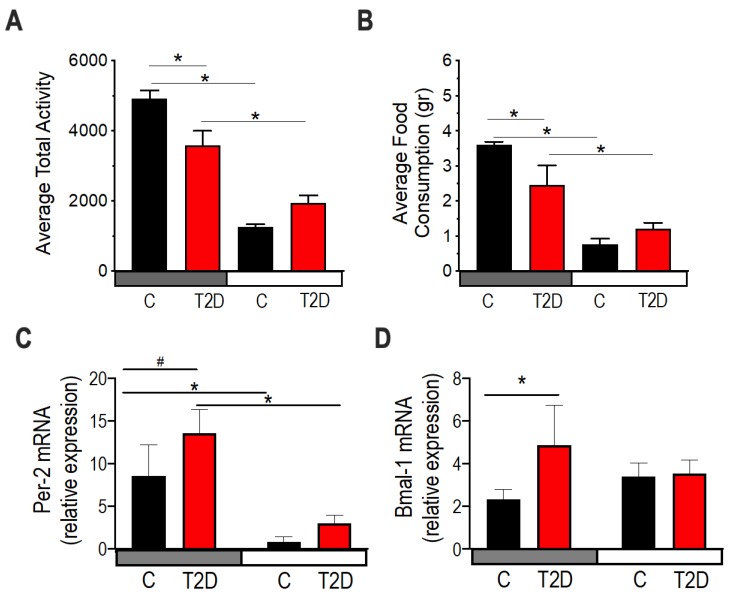
Diurnal activity, food consumption, and circadian gene expression in type 2 diabetic mice (T2D). 10-month-old db/db (red) and aged-matched controls, db/m (black). (**A**,**B**) All measurements were performed after a 48-h acclimation period followed by 48-h of data collection every 10 min. (**A**) Mean activity over the night period (19:00–07:00) and the day period (07:00–19:00). *n* = 8 per group. Two-way ANOVA. (*) indicate statistical significance at post-hoc multiple comparison test. (**B**) Food consumption during the dark period and the day. *n* = 8 per group. Two-way ANOVA. Asterisks indicate statistical significance at post-hoc multiple comparison test. (**C**) mRNA expression of Per-*2* in colonic tissue (*n* = 3). Two-way ANOVA. (*) indicate statistical difference *p* < 0.05, and (#) *p* < 0.01 in post-hoc comparisons. (**D**) mRNA expression of Bmal-1 in colonic tissue. (*n* = 3). Two-way ANOVA. (*) indicate statistical difference *p* < 0.05, and (#) *p* < 0.01 in post-hoc comparisons.

To examine why the diabetic microbiota were enriched in energy production pathways during the day, we examined the activity of the diabetic mice to determine whether they eat more food during the day, and thus, are feeding their microbiota. Average activity (Figure 6A) and food consumption (Figure 6B) in the day were not statistically different between control and diabetic mice. These data suggest that the loss of the oscillatory rhythms of the gut microbiota was not due to increased activity or altered feeding behavior during the resting phase but was due to other effects of diabetes.

Using RT-PCR, we examined two circadian genes Per2 and Bmal-*1* in the colon to determine if diabetes affects the host circadian clock. Per2 mRNA expression was significantly elevated in the night in both control and diabetic mice, albeit with a trend to be more elevated in diabetes (Figure 6C). Bmal-1 mRNA expression was significantly elevated in diabetic mice compared to controls (Figure 6D). Although we only tested the circadian genes at the transcription level and only at two times, these results suggest that the host clock gene expression is altered by diabetes, with possible implications for the physiology of the colonic tissue and its homeostasis with the gut microbiota.

## 4. Discussion

Recent advancements in microbiome research have featured a strong link between gut dysbiosis and diabetes, supporting that the gut microbiome modulates host metabolism [15]. Studies in people with type 2 diabetes show reduced members of the phylum Firmicutes and Clostridia, especially butyrate-producing (*Faecalibacterium prausnitzii*, *Roseburia intestinalis* and *Roseburia inulinivorans*) and increased members of Gram-negative betaproteobacteria (*g.Sutterela*), opportunistic pathogens (*Bacteroides caccae*, *Clostridium hathewayi*, *Clostridium ramosum*, *Clostridium symbosium*, *Eggerthela lenta* and *Esherichia coli*), sulphate-reducing bacteria (*Desulfovibrio*), *Lactobacillus* spp. (*Lactobacillus gasseri*, *Lactobacillus reuteri* and *Lactobacillus plantarum*), mucin degrading bacterium *Akkermansia municiphila*, and clusters of clostridia that are antagonistic to butyrate producing bacteria [14,15]. Previously, we examined whether intermittent fasting (IF) (every other day fasting) could prevent diabetic complications through effects on the microbiome in db/db mice. We found that IF restructured the gut microbiota of diabetic mice in a different direction compared to that of control mice, suggesting that host metabolism is important in shaping the microbiome responses to a nutritional stress [23]. However, we also observed that the ad-libitum groups had differential microbiome composition because of diabetes itself [23]. In that study, we did not detect the same bacterial species identified in people with type 2 diabetes, such as the aforementioned loss of butyrate producing bacteria, but we found alterations in families known to produce short-chain fatty acids (SCFAs), such as Lacnhospiracheae, and increased members of *Lactobacillus* spp. and *Akkermansia municiphila*. Our current study adds to these findings suggesting that not only is the composition of the bacteria altered because of diabetes, but also its temporal diurnal function. Diabetes is known to affect other aspects of circadian biology and physiology and this study showed that diabetes also affected the gut microbiota oscillations and functions.

Recent evidence suggests that bacterial oscillatory activity can regulate functions in distant tissues, such as the liver, having significant implications for host metabolism [19,20,21]. The expression of many metabolic liver genes, including genes involved in sterol biosynthesis, fatty acid, and linoleic acid metabolism, PXR and LXR activation, and methionine metabolism, are altered in the absence of a gut microbiome [19,21,33]. Ablation of the rhythmic oscillations of the gut microbiota by antibiotics, in concurrence with maintaining a normal feeding behavior, altered the diurnal oscillation of genes in the liver with some genes losing and others gaining de novo diurnal regulation [21]. Specifically, healthy mice treated with multi-spectrum antibiotics showed a loss of oscillatory rhythms of liver genes involved in oxidative phosphorylation, the TCA cycle and other catabolic pathways and a de novo gain of oscillatory rhythmicity in genes involved in liver amino acid and fatty acid metabolism [21]. Interestingly, we also found changes in plasma metabolites from these same pathways. However, while the liver oxidative phosphorylation and TCA cycle genes exhibited a loss of a diurnal pattern in the antibiotic-treated mice, we observed a gain of diurnal pattern in the TCA cycle and in mitochondrial functions in the blood from diabetic mice. These differences are likely due to cohort differences as we did not ablate the microbiome with antibiotics, but rather allowed a chronic disease to alter the microbiota oscillations. To investigate the true effects of the loss of microbiota oscillations in type 2 diabetes, we would have to deplete the microbiome in our diabetic cohorts and compare the oscillatory behavior of metabolic genes and metabolites in distant tissues and in the blood. This is a limitation of our study and must be considered when interpreting our findings and in attributing loss of oscillatory rhythms in the gut microbiota for changes in specific blood metabolites. Furthermore, luminal metabolites appear in the blood with a phase delay. In the current study, we did not measure the entire 24-h oscillatory rhythm of blood metabolites but rather examined only the two-time points within the 24-h period, one during the day and one during the night. Therefore, the timing of our blood samples may not accurately capture the changes that are due to specific bacteria, and caution should be taken when attributing specific metabolites as “biomarkers” for loss of gut microbiota oscillations.

Our results support the work of Leone et al. 2015 [33] showing that high fat diet abolished the circadian oscillations in the gut microbiota, especially of some members of the Lachnospiraceae family [33], who are potent short-chain fatty acid producers (SCFA) [34,35]. With high fat diet, oscillations of SCFA are lost, while oscillations of hydrogen sulfate are increased [33]. These metabolic oscillations impact the oscillations of circadian genes in the liver [20,33]. Unexpectedly, our global metabolomic analysis did not detect short-chain fatty acids in plasma. Nonetheless, we observed that many of the SCFA producers (family Lachnospiraceae, g. *Ruminococus*, g. *Coprococcus*, g. *Bifidobacterium* [36]) lost diurnal oscillations and other SCFA producers such as *Akkermansia municiphila* and g. *Prevotella* were either enriched or had a phase shift in their peaks. Proteobacteria (hydrogen sulfate producers) exhibited changes in the timing of their peak levels in diabetic mice compared to controls. Interestingly, pathway analysis in plasma from diabetic mice collected during the day compared to the night showed enrichment in the propanoate pathway. Propionate and butyrate can also be produced from lactate metabolism by many bacteria [37,38] and indeed, diabetic plasma had increased lactate concentrations. Finally, TCA cycle and lactate were among the pathways identified as gaining diurnal regulation suggesting that increased propionate or other short-chain fatty acids may be related to these changes, as propionate is implicated in increased mitochondrial function especially during exercise [38,39]. However, additional studies are required to test this hypothesis.

Among the metabolic pathways that showed an altered diurnal pattern in type 2 diabetes, we examined in detail the histidine, betaine, and methionine pathway because of their known involvement in vascular diseases. Several gut bacteria potentially involved in the histidine/histamine pathway including *Bifidobacterium*, *Clostridium*, *Bacteroides*, *Prevotella*, *Alercreutzia* [28] were also altered by diabetes. The histidine pathway is of particular interest because it shapes the host-microbiome immune responses, via suppression of the NLRP6 inflammasome signaling and anti-microbial peptide production, ultimately shaping the gut microbiota composition [40]. Moreover, production of imidazole propionate, which is part of this metabolic pathway has been implicated in insulin resistance [28]. The betaine and methionine metabolic pathway are also of interest for diabetic complications as this pathway produces trimethylamine oxide (TMAO), which is linked to increased cardiovascular risk and red meat consumption [41,42]. Several bacteria of the genus *Ruminococcus*, g. *Prevotella,* and some beta proteobacteria are associated with increased TMAO [42]. We observed increased levels of TMAO in diabetic mice, especially in the night and this may be related to the expanded concentrations of *Prevotella* observed at night. In contrast, Erypelotrichaceae, which are associated with reduced TMAO plasma concentrations [42], are reduced and lose their circadian regulation in the gut of the type 2 diabetic mice.

Finally, the methionine/homocysteine pathway also plays an important role in vascular complications because elevated levels of homocysteine have been linked to cardiovascular disease [41]. Homocysteine also leads to the elevated production of glutathione, a natural antioxidant likely as part of a compensatory mechanism. Studies comparing germ-free to conventional mice showed that the gut bacteria regulate glutathione and methionine metabolism [43] and many different bacterial species belonging to the families of Actinobacteria, Bacteroidetes, Bacilli, Clostridia, Erysipelotrichia, and to a greater degree proteobacteria are implicated in these pathways [44]. Our study shows that many of these families had altered diurnal phases because of diabetes. However, in order to establish a direct link between the changes in diurnal oscillation of bacteria and the plasma metabolic biotransformations, fecal transfer experiments with defined communities are required. 

The diurnal oscillatory rhythms of the gut microbiome are regulated, in part, by the host circadian clock [16,17], the composition of the diet [21,33], feeding [17], and sleeping schedule [17,45]. We did not observe any different activity and/or food consumption pattern during the night in diabetic mice compared to control mice. However, colonic Bmal-1 mRNA expression was elevated in diabetic mice indicating that host-disease associated factors such as circadian misalignment and/or hormonal and metabolic changes because of diabetes can affect the gut microbiome rhythms. Evidence exists that altered microbiota byproducts may affect the colonic and liver circadian gene expression as shown by studies demonstrating that polyamines regulate the period of circadian genes expression and depletion of polyamines with aging increase the circadian period [46].

Our research has several implications for the health of individuals with type 2 diabetes. First, we confirm that gut bacteria exhibit rhythmic oscillations and we show that the many members of the gut microbiome lose their rhythmicity in diabetes. Bacterial rhythmic oscillations regulate the host metabolism through the temporal production of bacterial metabolites that signal in critical metabolic pathways. Loss of the rhythmic oscillations of the microbiome may exacerbate the metabolic dysfunction in type 2 diabetes. Second, altered bacterial rhythmic oscillations may affect the efficacy of many anti-diabetic drugs. Bacterial oscillations regulate many liver functions including detoxification processes [21] making it possible that loss of bacterial oscillations could affect hepatic drug metabolism [21]. Many antidiabetic drugs affect the composition of the microbiome by inhibiting the growth of certain bacteria. For example, metformin action was shown to be mediated by depletion of *Bacteroides fragilis* resulting in an increase of glycoursodeoxycholic acid (GUDCA) and inhibition of FXR signaling. Thus, the timing of the administration of anti-diabetic drugs in combination with the type of formulation (short vs. sustained release) may influence drug efficacy. Gut bacteria have a large number of enzymes that degrade xenobiotics and drugs and can affect the absorption of anti-diabetic drugs [47]. In healthy controls, bacterial functions of xenobiotic and drug metabolism are enriched during resting or sleeping; however, we observed that this was not the case for diabetic mice.

It is possible to reestablish the lost diurnal bacterial oscillations by restricted feeding during the active period and by altering the diet composition. However, whether these strategies would help the type 2 diabetic microbiome remains to be tested. Our previous experience with intermittent fasting, where food is restricted every other day for 24 h, led to a differential restructuring of the gut microbiome in diabetic mice compared to control. That shows that the same nutritional intervention may result in very differently configurated microbiota communities in a diabetic versus a healthy host. A combination of restricted feeding within the active period and diet composition to correct dysbiosis may be a good strategy for the management of type 2 diabetes.

## 5. Conclusions

Overall, this was the first study to our knowledge examining the diurnal oscillations of the gut microbiota in type 2 diabetes. We found that in diabetic mice many members of the gut microbiota lost their diurnal oscillations, especially members of the Lachnosporaceae, Ruminococcaceae, Erysipelotrichaceae families, while members of the Prevotellaceae family, and the classes Actinobacteria and Proteobacteria exhibited significant phase shifts. During the night, the microbiota of controls was enriched in genes involved in xenobiotic and drug metabolism. In contrast, the microbiota of diabetic mice were enriched in genes involved in carbon and nitrogen metabolism. Analysis of the circulating metabolites showed alterations in the diurnal pattern of metabolic pathways known to be influenced by gut bacteria, such as the histidine, betaine, and methionine/cysteine pathway, mitochondrial function, and urea cycle. Altogether, the current study suggests that type 2 diabetes causes alterations in the rhythmic oscillations of the gut microbiota that can impact the host metabolome and the temporal regulation of metabolic pathways.

## Figures and Tables

**Table 1:** Statistical summary of the analysis of total 747 detected metabolites by UPLC-MS.

Significantly Altered Metabolites		Number of Metabolites (*p* ≤ 0.05)	Metabolites ( 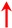 | 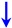 )
**Disease (Main effect)** **Time (Main effect)** **Interaction (Main effect)**		294 178 51	--- --- ---
Pair wise comparisons:			
Diabetes vs Control	at Day	234	170|64
Diabetes vs Control	at Night	189	134|55
Night vs Day	in Control	113	77|36
Night vs Day	in Diabetes	130	70|60

**Table 2:** Enrichment analysis of predicted pathways for bacterial functions that were different in day vs night in control and type 2 diabetic mice.

**Day vs. Night in Control—Enrichment Analysis**					
**Pathway**	**Total**	**Expected**	**Hits**	**Pval**	**FDR**
**Zeatin biosynthesis**	3	0.0202	1	0.0201	1
**Nitrotoluene degradation**	7	0.0472	1	0.0463	1
**Caffeine metabolism**	9	0.0607	1	0.0592	1
**Naphthalene degradation**	17	0.115	1	0.109	1
**Linoleic acid metabolism**	20	0.135	1	0.127	1
**Drug metabolism - other enzymes**	20	0.135	1	0.127	1
**Chloroalkane and chloroalkene degradation**	23	0.155	1	0.145	1
**Various types of N-glycan biosynthesis**	28	0.189	1	0.174	1
**Folate biosynthesis**	29	0.196	1	0.179	1
**Fatty acid degradation**	39	0.263	1	0.234	1
**Day vs. night in Diabetes – Enrichment Analysis**					
**Carbon metabolism**	249	4.14	11	0.00167	0.247
**Pyruvate metabolism**	74	1.23	4	0.0326	1
**Zeatin biosynthesis**	3	0.0499	1	0.0491	1
**Citrate cycle (TCA cycle)**	53	0.882	3	0.0562	1
**2-Oxocarboxylic acid metabolism**	57	0.949	3	0.0672	1
**Biosynthesis of amino acids**	222	3.7	7	0.069	1
**Carbon fixation pathways in prokaryotes**	60	0.999	3	0.0759	1
**Synthesis and degradation of ketone bodies**	5	0.0832	1	0.0806	1
**Lysine degradation**	30	0.499	2	0.0876	1
**Amino sugar and nucleotide sugar metabolism**	64	1.07	3	0.0884	1

## Supplementary Materials

The following are available online at https://www.mdpi.com/2072-6643/11/10/2310/s1, Figure S1: Diurnal oscillatory rhythms of the gut microbiota inT2D at the family (A) class (B) and (C) phylum level.
